# Progress in Medicine

**Published:** 1896-02-08

**Authors:** 


### Progress in Medicine.

### GOUT AND RHEUMATISM.

**Gout.**—The occurrence of acute attacks in other situations than the joints is discussed by Jaccoud[1] in an interesting paper, in which he gives the history of a patient who suffered for nineteen years from regular articular gout. He was then seized with violent dyspnœa, which was actively treated on the supposition that it was of renal origin, improvement followed, but suddenly the dyspnœa returned with a paroxysmal cough and slight rise of temperature. Next day an acute attack of gout in several joints appeared, and the dyspnœa vanished simultaneously. Similar outbreaks of gastralgia, hepatalgia, or other affections may replace an expected gouty paroxysm; and, on the other hand, an outbreak of articular gout may subside, and be at once followed by cardiac, pulmonary, or cerebral manifestations. Finally, as Jaccoud points out, besides these "alternating" and "metastatic" forms, we may meet with a third type, or "masked gout," consisting of various irregular affections in the subjects of hereditary gout, who have not themselves ever suffered from a definite attack. Plumbic gout is specially marked by the frequency and early development of serious visceral phenomena. Common as this form of gout is in England, Jaccoud finds it more rarely in France, where it probably does not cause 10 per cent. of the disease, while in Germany it is still rarer. Though, as we have said, it is soon followed in many cases by visceral complications, it begins by an acute articular attack, and is not preceded by the long train of lithæmic symptoms so common in hereditary gout.

The uric acid controversy, owing to recent discoveries, is becoming an acute one, and the tendency of the following papers is to show that though an excess of uric acid is present in gout, some other substance is the active agent in the inflammation of the joints, the uric acid being passively deposited in them, just as lead, bacteria, and other foreign substances are deposited in the neighbourhood of any accidental lesion. Kolisch[2] finds that the destruction of white corpuscles yields not only uric acid but also the "alloxuric" bases. In health they are all converted into the former substance and excreted, but when the kidneys fail the acid is largely replaced by these xanthic or alloxuric substances. The essence of the disease is the increased disintegration of nucleo albumens and the increased formation of alloxuric bases. As to treatment, we have no remedy which checks the disintegration, but we should avoid giving sweetbread and other glands, alcohol, meat extracts, and asparagus, neither should we permit violent exercise.[3] Alkalies act only by stimulating the kidneys. A diagnosis of gout can be made if we find in the urine an increase of alloxuric substances or the perinuclear basophilia of Neusser. Klemperer,[4] on the other hand, believes that in gout some unknown substance leads to an inflammatory and necrotic process in various tissues; the necrotic parts have the power of attracting from the blood uric acid which is present in excess. This is much the same as the view of Von Noorden, but Klemperer has made careful analyses of the blood of patients suffering from gout and other diseases, and of healthy persons; and while he confirms Garrod's theory that the blood is abnormally rich in uric acid during gout, he shows that there is an equal excess in other diseases. But from the results he has obtained by another series of experiments he strongly denies Garrod's position that there is a diminished excretion of uric acid in gout, and in additional support of his view points to the fact that the administration of thymus gland in gout causes a not less increased elimination of uric acid than is seen if the gland is given to a healthy person. Hence the power of excretion in gout is not diminished, and the excess of uric acid in the blood is due to increased production and not to lessened excretion. Again, this excess is not the cause of the deposit, for the blood is so far from saturated that in his experiments the blood serum of acute gout could dissolve as much or more than the serum of healthy persons. Finally, the lessened alkalinity is not a cause, for it does not equal that observed in other diseases without the slightest symptom of gout. On the whole, he argues that some unknown agent produces the change in the tissues, which then have such a chemical affinity for the uric acid that the blood is unable to dissolve it again. He would direct treatment rather to the healing of the damaged tissues than to increasing the solvent power of the blood, which is already sufficient. This he would do by increasing oxidation and excretion. Muscular work, diuretics, baths, and a frugal diet are therefore recommended. In the discussion of a kindred subject Ritter[5] raises the question why urates are deposited in the water of persons where neither the degree of acidity, concentration or percentage of uric acid are abnormally high, and finds from experiments that crystalline or brickdust deposit depends either on a total absence or a relatively insufficient quantity of di-sodic phosphates.

**Rheumatism.**—Since the papers of Newsholme on the epidemic and climatic characters of acute rheumatism little has been effected towards the discovery of its active cause; many observations tend rather to show that various agents may produce it, and that there are indeed several clinical varieties of disease which we group under the name. Chvostek,[6] after getting negative bacterial results from an examination of the urine, argues that the joint lesions are due not to the direct action of bacteria, but to the effects of toxic substances such as is seen in the results of the injection of diphtheria anti-toxin. The toxine may be produced, for instance, in the intestinal tract by bacteria not otherwise pathogenic, or the tonsils may be the seat of elaboration. The disease would thus be of the nature of a local intoxication process rather than of a septicæmic character. J. Heidenhain[7] draws attention to the monoarticular cases, which are not infrequent. They yield rapidly to salicylates, but there is usually no febrile temperature. The shoulder is most often affected, and occasionally permanent injury to movement follows. Are these only a mild form of the acute disease, and is it also productive of cardiac lesions? Robin[8] contends that lumbago is really an articular affection, that the points of greatest pain are in the median line over the vertebræ or else over the sacro iliac junctions, that the patient prefers to stand in a position which would put the erecter muscles in pain if they were affected, while this slightly forward position does best shield the joints. He recommends an infusion of the leaves of jaborandi to be taken in the morning, and in chronic cases repeated in a day or two. He is careful to warn his readers against the use of the remedy when heart disease is present. Gamgee[9] published recently an important paper on synovitis and suppurative arthritis as complications of cutaneous erysipelas which has been rarely discussed in England. He collected twenty-nine cases, and divides attacks into those where the erysipelatous blush involves the skin over the joint, and those where the affected joint is distinct from the erysipelas. In the first class the infection reaches the joint along the lymph channels by continuity of tissue, but in the second we have a true pyæmia, and he shows that the streptococcus erysipelatosis can and does produce in these ways suppurative arthritis. The prognosis in the cases where the joint is distant from the erysipelas is very much more grave than in the others; seven where the joint were thus situated were all fatal. [An account of similar lesions in the rheumatism of scarlet fever among 7,000 patients in the North-Western Fever Hospital was given in The Hospital of January 11th.]

R. Caton[10] has pursued for thirteen years an inquiry into the history and treatment of rheumatic endocarditis, which extended over three hundred cases. He found the opinions of modern authorities most conflicting, and finally adopted a treatment in which the percentage of heart lesions was reduced. His plan was to keep the patient in prolonged absolute rest in bed, the body clothed in flannel, with a milk diet, salicylates, cholagogues, and small blisters on those joints where the pain failed to disappear. As soon as the slightest "woolliness" of a heart sound was heard, ten grains of iodide was given three times a day with the salicylates and a blister of the size of a florin was placed over the apex of the heart. A second is placed close to its site as soon as the irritation caused by it is subsiding, and others in succession. In a week or ten days the murmur gradually subsides, though sometimes this takes some weeks, during which absolute rest is maintained. Thirty-nine successful cases averaged thirty-six days each in bed. He regards the blisters as acting by reflex stimulation of the trophic nerves of the heart, and has elsewhere shown that the stimulation of cutaneous nerves does alter the diameter of arterioles and the electrical potential (as measured by the galvanometer) in abdominal organs which correspond to the area stimulated. However this may be, any treatment which lessens the amount of heart disease following upon rheumatic fever is of the highest importance.

Mikhaline[11] reports three cases of obstinate sciatica, in which treatment by nitro-glycerine was of great benefit. One drop of the official solution was given three times a day and slightly increased as the patient became used to it. The injection of a minim of guiacol dissolved in ten of chloroform over the course of the nerve has also been recommended warmly for the same affection.

[1] Med. Week, Oct. 18. [2] Lancet, Nov. 9. [3] Med. Week, Oct. 25. [4] Med. Chron., Nov. [5] Am. Med. Surg. Bulletin, Sep. 15. [6] Am. J. Med. Sc., Oct. [7] Am. J. Med. Sc, Nov. [8] N. Y. Med. Rec., Oct. 26. [9] Birm. Med. Rev., Sep. [10] Med. Rec., Nov. 23. [11] Med. Rec., Nov. 23.

